# 基于醛-胺冷冻聚合反应的亚胺类共价有机框架整体材料的制备

**DOI:** 10.3724/SP.J.1123.2026.06001

**Published:** 2026-11-08

**Authors:** Luyao DING, Lin ZHU, Guoli QIAO, Zhongshan LIU, Ruixia GAO

**Affiliations:** 1. 陕西师范大学化学化工学院，陕西 西安 710119; 1. School of Chemistry and Chemical Engineering，Shaanxi Normal University，Xi’an 710119，China; 2. 西安交通大学化学学院，陕西 西安 710049; 2. School of Chemistry，Xi’an Jiaotong University，Xi’an 710049，China

**Keywords:** 共价有机框架, 整体材料, 冷冻聚合, 通孔, covalent organic frameworks （COFs）, monolith, cryopolymerization, through-pore

## Abstract

亚胺类共价有机框架材料作为色谱柱分离固定相和样品预处理材料备受关注，但其形貌和形态调控仍面临挑战，严重限制了该类材料在分离分析领域的实际应用。近期研究发现，醛-胺聚合反应可以在低温冷冻条件下进行，且以1，4-二氧六环为溶剂冷冻形成的松针状晶体可构筑微米级的定向通孔；然而该模板的规整性有待提升。在此基础上，本研究提出以二甲基亚砜为醛-胺聚合反应体系的新型冷冻模板剂，利用其在低温下形成的针状晶体构筑更为规整的定向通孔结构，进而发展了一种基于醛-胺冷冻聚合反应制备亚胺类共价有机框架整体材料的新方法。首先在室温下配制单体质量分数为5%的均相预聚溶液，然后在液氮条件下使二甲基亚砜溶剂定向冷冻形成晶体模板；多元醛单体与多元胺单体聚集在晶体模板之间的缝隙中；将体系转移至-18 ℃进行聚合反应，形成无定形聚合物；最后通过晶化处理，该聚合物转化为共价有机框架整体材料。扫描电子显微镜图、粉末X射线衍射谱图和氮气物理吸附数据表明，该晶态框架整体材料具有微米级的定向通孔和纳米级的有序孔道；相比于1，4-二氧六环晶体模板所制备的整体材料，二甲基亚砜晶体模板诱导构筑的定向通孔结构更规整，通孔内壁光滑。进一步采用不同的醛-胺聚合反应体系，验证了“先冷冻成型，再高温晶化”的两步法成型策略制备共价有机框架整体材料的普适性，为推动该类材料在分离分析领域中的应用提供了有益借鉴。

## Abstract (en)

Imine-linked covalent organic frameworks （COFs） have attracted considerable attention as stationary phases for chromatographic separation and as sample pretreatment materials，owing to their exceptional properties including high specific surface areas，well-defined nanopores，and excellent thermal and chemical stability. However，the practical deployment of COFs in separation science is still hampered by the limited control over their macroscopic morphologies and microscopic architectures，especially when they are constructed as monolithic materials that require continuous through-pores to facilitate rapid mass transfer and low backpressure. In our previous work，we discovered that the aldehyde‑amine polycondensation reaction could proceed under cryogenic conditions，and that using 1，4‑dioxane as the solvent led to the formation of pine needle‑like crystals upon freezing. These crystals acted as sacrificial templates to create unidirectional macroporous channels within the resulting COF monoliths. Nevertheless，the structural regularity of these channels was less than ideal. This motivated us to search for alternative solvents that could form more regular crystal templates. In this work，we propose dimethyl sulfoxide （DMSO） as a new template for the aldehyde‑amine polymerization system. We found that DMSO，when subjected to unidirectional freezing，forms highly uniform needle‑shaped crystals that are different from the pine‑needle morphology of 1，4‑dioxane crystals. These DMSO crystals provide a more orderly template，guiding the monomer assembly into a well‑aligned framework. Based on this finding，we have developed a novel two‑step approach for preparing imine‑linked COF monoliths. Briefly，a homogeneous prepolymerization solution containing 5% （mass fraction） total monomers （a combination of multi‑aldehyde and multi‑amine building blocks） in DMSO is prepared at room temperature. The solution is then cast into a mold and subjected to unidirectional freezing in liquid nitrogen，which forces the DMSO to crystallize in an oriented manner，forming needle‑like crystals. During this process，the monomers are excluded from the growing crystals and concentrated in the narrow gaps between them. The frozen system is then transferred to a -18 °C cryostat and allowed to react for a certain period，during which the monomers polymerize into an amorphous polymer. After washing to remove DMSO and drying under vacuum，the obtained amorphous monolith is subjected to a crystallization treatment in a suitable solvent mixture under solvothermal conditions，which triggers the structural rearrangement into crystalline COFs，while preserving the macroscopic channel architecture. The resulting COF monoliths were characterized by scanning electron microscopy （SEM），powder X‑ray diffraction （PXRD），and nitrogen physisorption measurements. SEM images show well‑oriented，unidirectional macroporous channels with diameters in the micrometer range. Compared with the materials prepared using 1，4‑dioxane templates，the channels obtained with DMSO are significantly more regular，with smoother inner walls. PXRD patterns confirm the crystallinity of COF monoliths. The Brunauer-Emmett-Teller （BET） surface areas are comparable with those of powdery materials prepared by conventional solvothermal methods，and those of monoliths obtained with 1，4-dioxane templates. We finally demonstrated the versatility of our “freeze-shaping followed by crystallization” strategy by using different aldehyde-amine monomer pairs. The two-step strategy offers a general platform for shaping COFs into monolithic forms with desired architectures，which is expected to enhance mass transfer and separation efficiency. This work provides valuable insights into morphology engineering of COFs and is anticipated to promote their practical application in high‑performance liquid chromatography，solid‑phase extraction，and other separation technologies.

亚胺类共价有机框架（covalent organic frameworks，COFs）是由多元醛单体与多元胺单体通过缩合反应而形成的晶态多孔材料。亚胺类COFs材料的合成条件相对成熟，单体种类多，具有较好的化学稳定性、可调的有序孔道及较高的比表面积，近年来作为气相色谱固定相^［1-4］^、液相色谱固定相^［5-8］^、固相萃取和吸附富集材料^［9-13］^等备受关注。例如，Yang等^［2］^将TpBD-COFs微球涂覆于毛细管柱内壁，实现了对正构烷烃、环己烷/苯、*α*-蒎烯/*β*-蒎烯及醇类等混合物的气相色谱分离分析。研究人员进一步将手性中心与COFs材料相结合^［3，4］^，所制备的手性毛细管柱，对1-苯乙醇、柠檬烯、香茅醛和茴香酮等对映体表现出优异的拆分能力。Huang等^［5］^将Salen-COFs涂敷在硅球表面，制备了填充柱，结果表明三维微孔通道对邻二甲苯具有优先识别特征，从而实现了二甲苯异构体的基线分离。在样品前处理领域^［9-11］^，亚胺类COFs材料作为预富集材料，与目标分析物存在疏水、*π-π*、氢键及范德华力等多重相互作用模式，且其纳米孔对大分子具有尺寸排阻效应，从而能够实现小分子目标物的选择性富集。

尽管COFs在分离分析领域潜力巨大，但其实际应用仍面临两个核心瓶颈：一是传统溶剂热法合成的COFs多为不溶性、不规则颗粒粉末，难以加工成色谱柱所需的连续固定相；二是COFs尺寸、形状、通孔结构的精准调控依然困难。在色谱柱分离中，固定相的形貌均匀性和颗粒尺寸直接影响柱效和分离度。例如，Wang等^［14］^系统研究了单晶COFs的粒径对气相色谱分离性能的影响，发现随着单晶COFs粒径从0.4 μm增大至1.6 μm，二甲苯异构体的分辨率从2.55下降至1.23。在固相萃取和吸附应用中，不规则形貌和较宽的粒径分布不仅会降低涂层或填充柱的重现性，还会限制目标分析物向孔道内部活性位点的快速扩散。

在传统溶剂热法合成亚胺类COFs材料的过程中，将单体与乙酸催化剂混合时会发生剧烈的聚合反应，先形成无定形聚合物，然后在高温条件下转变为晶态框架材料，而无定形聚合物在很大程度上决定了最终亚胺类COFs的粉末形态和无规则形貌^［15］^。相比之下，无催化剂的醛-胺聚合反应和痕量乙酸催化的醛-胺冷冻聚合反应的速率缓慢可控^［16-18］^，为材料形态和形貌的调控提供了有利条件，且能够利用聚合诱导相分离法或模板法构建具有通孔结构的亚胺类COFs整体材料。本课题组^［17］^发现，多元醛单体与多元胺单体在-18 ℃低温条件下也能发生聚合反应，该温度低于1，4-二氧六环的凝固点（12 ℃），因此溶剂以晶体形式存在；首先以1，4-二氧六环晶体为模板合成具有定向通孔结构的无定形聚合物整体材料，然后通过晶化处理得到多级孔COFs整体材料。醛-胺冷冻聚合反应方法所制备的湿态COFs整体材料经简单的真空干燥后仍能保留微米级的通孔结构，避免了超临界二氧化碳干燥和冷冻干燥过程。

醛-胺冷冻聚合反应采用的溶剂需具备较好的单体溶解能力，可形成均相预聚溶液；同时具有较高的凝固点，在-18 ℃的聚合反应温度下以固体形式存在，从而可作为模板构建定向通孔结构。1，4-二氧六环作为模板剂，使得聚合反应与晶化所需要的溶剂体系具有较好的兼容性，但其在冷冻条件下形成的晶体模板规整性不理想。二甲基亚砜（DMSO）也是常用的良溶剂，凝固点为18.5 ℃，在低温条件下形成光滑的针状晶体，与松针状的1，4-二氧六环晶体存在显著差异（图1a），为提高COFs整体材料的定向通孔的规整性和调控通孔内壁结构提供了有利条件。基于此，本研究以DMSO为溶剂和冷冻模板，首先利用醛-胺冷冻聚合反应制备具有定向通孔结构的无定形整体材料；然后经过洗涤干燥，在常用的溶剂热条件下进行晶化处理，制备晶态COF整体材料。采用均苯三甲醛（TFB）、对苯二甲醛（TPA）、对苯二胺（PDA）、1，3，5-三（4-氨基苯基）苯（TAPB）、4，4′，4″，4‴-（乙烯-1，1，2，2-四基）四苯胺（ETTA）和四（4-氨基苯基）甲烷（TAM）合成了TFB-PDA、TAPB-TFB、ETTA-TPA、TAM-TPA等4种亚胺键连接的无定形聚合物整体材料，通过晶化处理，获得了COF-LZU1（LZU： Lanzhou University）、TAPB-TFB COF和ETTA-TPA COF，验证了“先冷冻成型，再高温晶化”的两步法成型策略制备共价有机框架整体材料的普适性，为新型色谱分离和样品前处理材料的简单高效制备奠定基础。

**图1:**
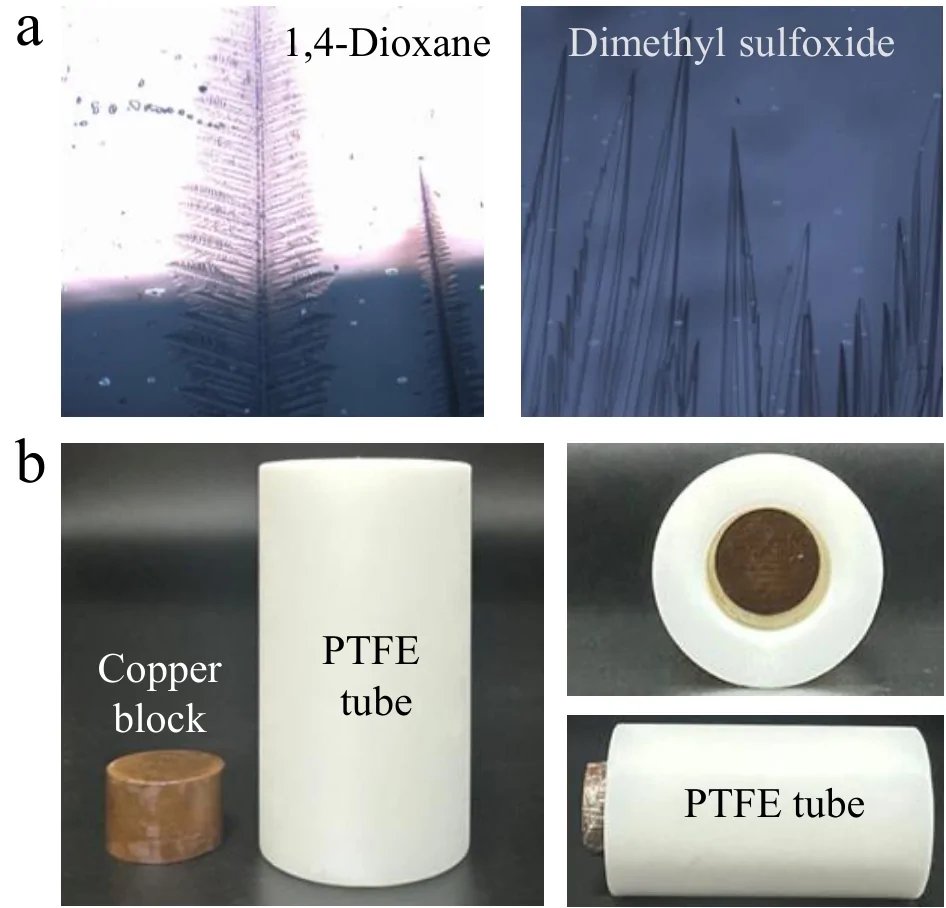
（a）金相显微镜下1，4-二氧六环和二甲基亚砜定向冷冻形成的晶体形貌对比和（b）实验室自制的冷冻聚合反应容器 a. The crystal images were recorded by a Zeiss Axio Scope A1 microscope. b. copper block： diameter，15.0 mm； height，10.0 mm； PTFE （polytetrafluoroethylene） tube： height，48.5 mm； inner diameter，15.0 mm； outer diameter，27.0 mm.

## 1 实验部分

### 1.1 仪器、试剂与材料

粉末X射线衍射仪（Bruker D8 X，布鲁克AXS有限公司，德国），扫描电子显微镜（SEM，TM3000，日立高新技术公司，日本），氮气物理吸附仪（Micromeritics ASAP 2460，麦克默瑞提克公司，美国），金相显微镜（Axio Scope A1，卡尔·蔡司显微镜有限公司，德国）。实验室自制的定向冷冻聚合反应容器由聚四氟乙烯（PTFE）管和圆柱形铜块组合（图1b，下文称为PTFE反应釜），该容器放在液氮中的大铜块表面，从而实现预聚溶液的定向冷冻。

TFB（97%）、PDA（97%）、TAPB（98%）、ETTA（95%）和TAM（97%）购自郑州阿尔法化工有限公司；无水DMSO（99%）、TPA（98%）、1，4-二氧六环、均三甲苯和冰醋酸（HAc）购自梯希爱化成工业有限公司；乙醇和正己烷购自国药集团化学试剂有限公司。采用HAc和DMSO配制HAc浓度为0.01 mol/L或0.1 mol/L的溶液，分别记作DMSO-HAc-0.01和DMSO-HAc-0.1。

### 1.2 COF-LZU1整体材料的合成

在离心管A中加入PDA（48.7 mg，0.45 mmol）和DMSO-HAc-0.01溶液（0.68 mL），在离心管B中加入TFB（48.6 mg，0.30 mmol）和DMSO-HAc-0.01溶液（1.00 mL）；经超声处理1 min，离心管A中的单体溶解形成澄清溶液，离心管B中的单体形成悬浊液。将离心管A中的溶液加入离心管B中，超声处理至形成均相预聚溶液，将该溶液转移至底座为铜块的反应容器中，并在液氮条件下进行定向冷冻。将冷冻状态的反应体系在-18 ℃冰箱中反应48 h，然后转移至4 ℃冰箱继续反应24 h。依次采用乙醇（4×10 mL）和正己烷（4×10 mL）对所生成的固体材料进行洗涤，然后将其置于60 ℃的真空烘箱中干燥12 h，得到无定形的TFB-PDA聚合物整体材料。将该整体材料、1，4-二氧六环（3.00 mL）和醋酸水溶液（0.60 mL，3 mol/L）加入容积为25 mL的PTFE反应釜中，密封后在120 ℃条件下晶化72 h；依次采用乙醇（4×10 mL）和正己烷（4×10 mL）洗涤整体材料，将其置于60 ℃的真空烘箱中干燥12 h，得到晶态COF-LZU1整体材料。

### 1.3 TAPB-TFB COF整体材料的合成

在离心管A中加入TAPB（70.3 mg，0.20 mmol）和DMSO-HAc-0.01溶液（0.78 mL），在离心管B中加入TFB（32.4 mg，0.20 mmol）和DMSO-HAc-0.01溶液（1.00 mL）；经超声处理1 min，离心管A中的单体溶解形成澄清溶液，离心管B中的单体形成悬浊液。将离心管A中的溶液加入离心管B中，超声处理至形成均相预聚溶液，将该溶液转移至底座为铜块的反应容器中，并在液氮条件下进行定向冷冻。后续处理同1.2节，得到晶态TAPB-TFB COF整体材料。

### 1.4 ETTA-TPA COF整体材料的合成

在离心管A中加入TPA（40.2 mg，0.30 mmol）和DMSO-HAc-0.1溶液（0.78 mL），在离心管B中加入ETTA（58.9 mg，0.15 mmol）和DMSO-HAc-0.1溶液（1.00 mL）；经超声处理1 min，离心管A中的单体溶解形成均相溶液。针对该体系中ETTA溶解性较差的问题，将离心管A中的溶液加入离心管B后，通过80 ℃水浴与超声交替的短时间处理，可以获得均相预聚溶液，将该溶液转移至底座为铜块的反应容器中，并在液氮条件下进行定向冷冻。后续处理同1.2节，得到无定形的ETTA-TPA聚合物整体材料。将该整体材料、1，4-二氧六环（5.12 mL）、均三甲苯（1.28 mL）、冰醋酸（1.90 mL）和水（1.30 mL）加入容积为25 mL的PTFE反应釜，密封后在70 ℃条件下晶化24 h；依次采用乙醇（4×10 mL）和正己烷（4×10 mL）洗涤整体材料，然后将其置于60 ℃的真空烘箱中干燥12 h，得到晶态ETTA-TPA COF整体材料。

### 1.5 TAM-TPA聚合物整体材料的合成

在离心管A中加入TPA（40.2 mg，0.30 mmol）和DMSO-HAc-0.01溶液（0.78 mL），在离心管B中加入TAM（60.0 mg，0.15 mmol）和DMSO-HAc-0.01溶液（1.00 mL）；经超声处理1 min，离心管A中的单体溶解形成均相溶液，将其加入离心管B后，超声处理至形成均相预聚溶液，将该溶液转移至底座为铜块的反应容器中，并在液氮条件下进行定向冷冻。后续处理同1.2节，得到无定形的TAM-TPA聚合物整体材料。将该整体材料、1，4-二氧六环（1.60 mL）和醋酸水溶液（0.20 mL，6 mol/L）加入25 mL的PTFE反应釜，密封后在70 ℃条件下晶化24 h；依次采用乙醇（4×10 mL）和正己烷（4×10 mL）洗涤整体材料，然后将其置于60 ℃的真空烘箱中干燥12 h。

## 2 结果与讨论

### 2.1 冷冻聚合反应方法与晶化处理

在前期工作^［17］^中，基于1，4-二氧六环晶体为致孔模板的制备方法中优化的单体质量分数为5%、醋酸催化剂用量为0.01 mol/L或0.1 mol/L。因此，在本研究中采用以上优化条件，将4种聚合反应体系中的单体质量分数设定为5%，重点讨论以DMSO晶体为致孔模板方法的可行性及普适性。1，4-二氧六环是传统溶剂热法合成亚胺类COFs材料常用的溶剂，其作为模板得到的无定形聚合物无需洗涤干燥过程，可直接进行晶化处理。相比之下，DMSO溶剂晶体作为模板得到无定形聚合物整体材料后，为避免DMSO对溶剂热条件下无定形转变为晶态框架的影响，需要对无定形聚合物整体材料进行溶剂置换或干燥，重新将其浸入合适的溶液中进行晶化处理。

### 2.2 COF-LZU1整体材料的制备和表征

以COF-LZU1体系为例，整体材料的定向通孔和有序孔道的构筑过程如图2所示。均相预聚溶液在定向冷冻条件下得到固体，该固体在-18 ℃下通过醛-胺聚合反应形成具有定向通孔结构的无定形聚合物整体材料；该整体材料经过晶化处理，保留了定向通孔结构，同时其骨架转变为具有有序孔结构的框架。

**图2:**
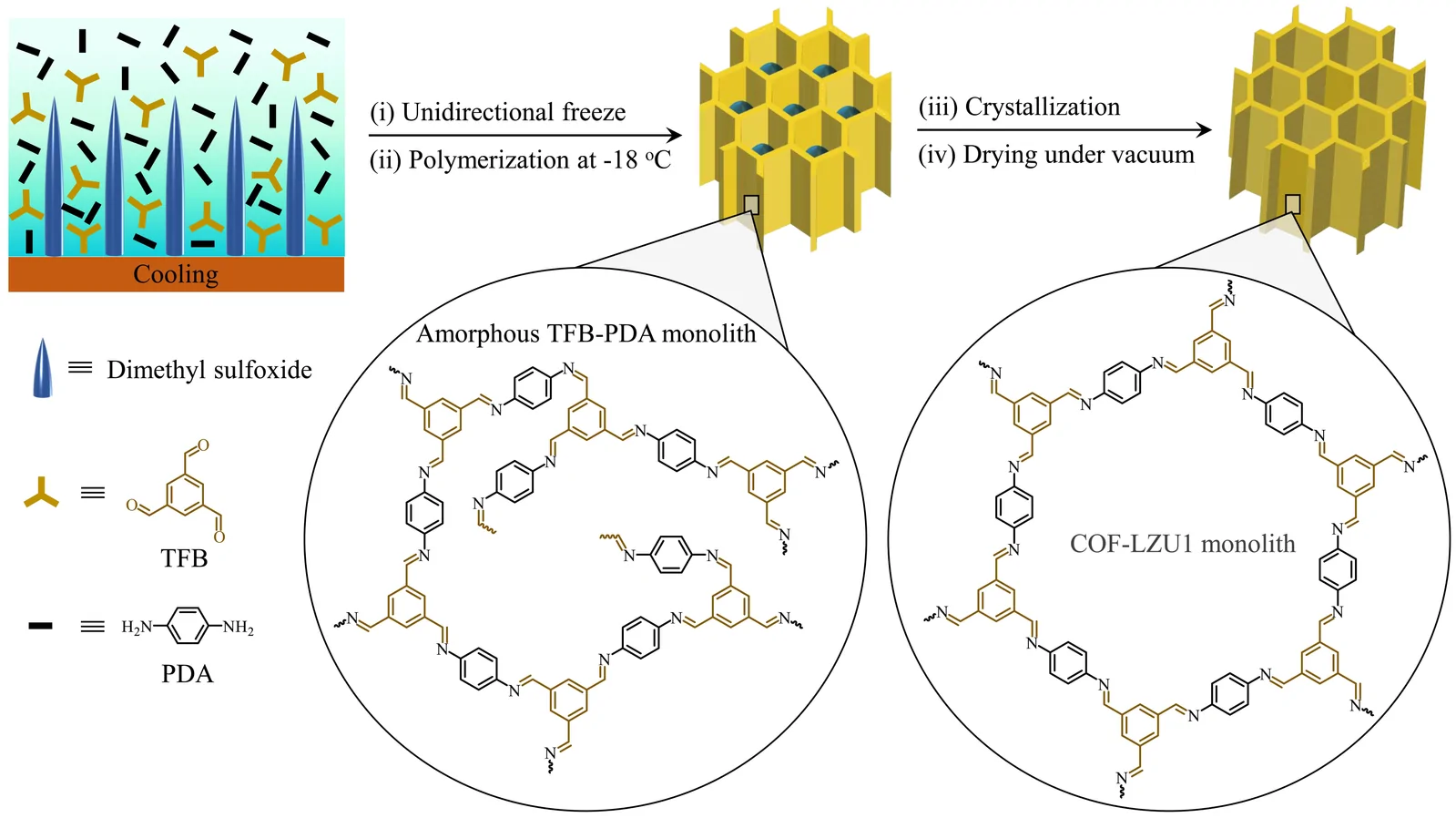
基于醛-胺冷冻聚合反应的COF-LZU1整体材料制备示意图

图3a是冷冻聚合反应所制备的TFB-PDA无定形聚合物整体材料的实物图，真空干燥后材料形态保持完好，其直径为14 mm，以冷冻聚合反应容器的内径（15 mm）为标准，收缩率小于7%。图3b是TFB-PDA无定形聚合物整体材料的SEM图，呈现规则的定向通孔结构。经过晶化处理后，COF-LZU1整体材料保留了定向通孔结构，同时在孔壁上出现毛刺结构（图3c），可能是高温下基于可逆亚胺键的骨架重组导致的，这一现象在采用无定形到晶态的固相转变方法制备COFs材料过程中较为常见^［19］^。与使用1，4-二氧六环相比，DMSO溶剂模板所制备整体材料的定向通孔更均匀，内壁光滑（图3d），与DMSO溶剂晶体的形貌一致（图1a）。图3e和图3f分别是根据COF-LZU1整体材料的SEM图像统计分析得到的其通孔尺寸分布和骨架尺寸分布，定向通孔尺寸的平均值为（45.82±9.61） μm，通孔骨架的壁厚平均值为（2.42±1.02） μm。

**图3:**
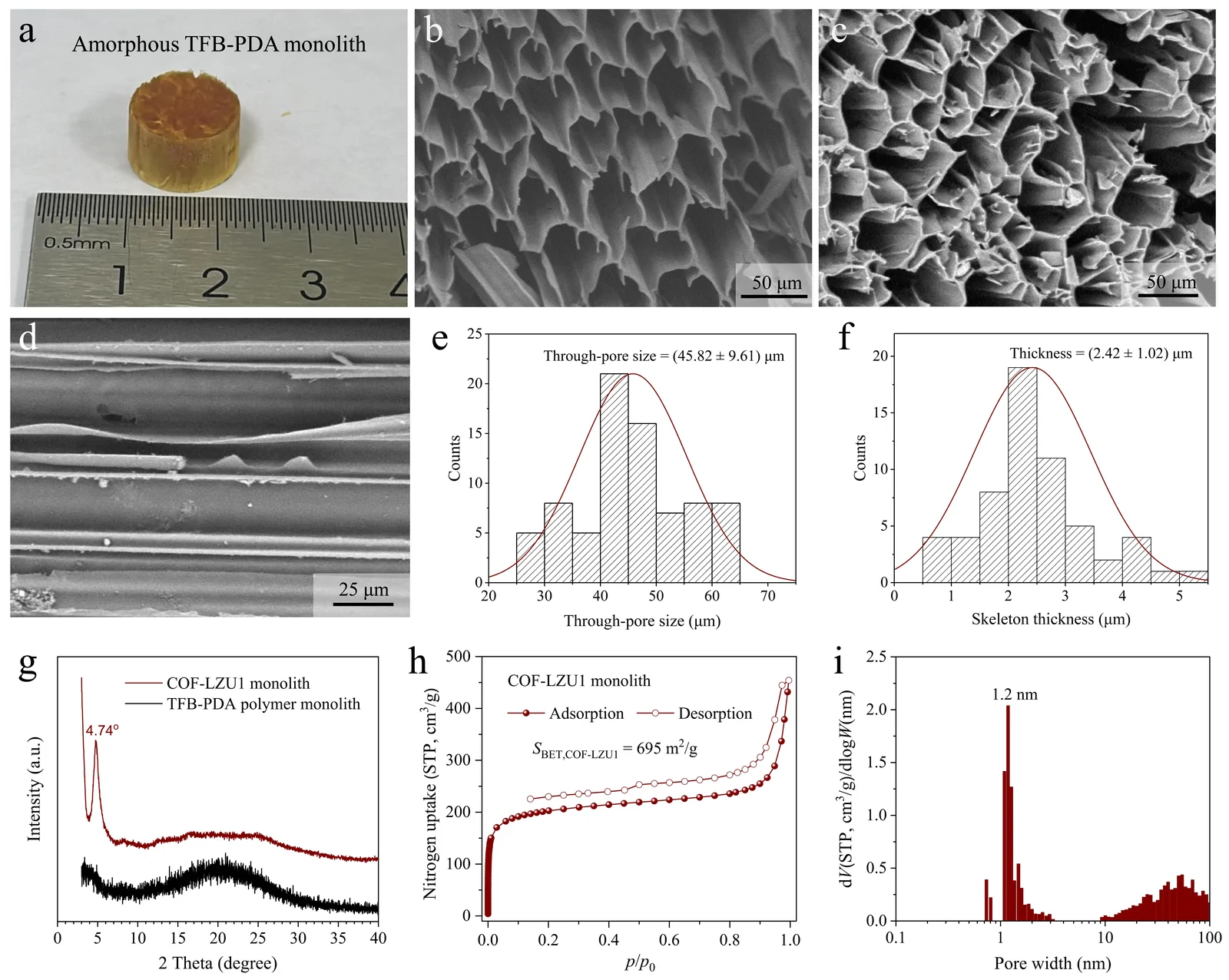
无定形聚合物TFB-PDA整体材料的（a）实物图和（b）SEM图；COF-LZU1整体材料的（c）截面和（d）通孔内壁SEM图、（e）通孔尺寸分布、（f）骨架尺寸分布、（g）粉末X射线衍射谱图、（h）氮气物理吸附等温线和（i）有序孔径分布

图3g是COF-LZU1整体材料的粉末X射线衍射谱图，在2*θ=*4.74^o^处出现（100）的特征衍射峰，与文献［^20^］报道一致，验证了“先冷冻成型，再高温晶化”策略制备晶态框架整体材料的可行性。随后，采用氮气物理吸附仪对COF-LZU1整体材料的孔结构进行了表征，如图3h和图3i所示，根据Brunauer-Emmett-Teller模型计算得出材料的比表面积为695 m^2^/g，其数值高于采用1，4-二氧六环为溶剂所制备的COF-LZU1粉末材料（410 m^2^/g）^［20］^和整体材料（621 m^2^/g）^［17］^，根据非定域密度泛函理论（non-local density functional theory，NLDFT）模型得到材料骨架内的有序孔径为1.20 nm，是COF-LZU1材料的特征孔径。综上所述，所制备的COF-LZU1整体材料具有典型的多级孔结构，且其微米级的骨架壁厚有利于提高目标物的传质效率。

### 2.3 基于冷冻聚合反应的COFs整体材料制备方法的普适性

为验证基于冷冻聚合反应的COFs整体材料制备方法的普适性，本研究选择了3种较具代表性的材料体系，如图4所示。相比于上述的TFB-PDA体系，TFB与TAPB的聚合反应速率更快、交联度更高；而TPA与ETTA是制备具有异孔结构的共价有机框架材料的典型体系；TPA与TAM是制备具有三维拓扑结构的共价有机框架材料的常见体系。所有体系的均相预聚溶液中单体质量分数均为5%，其中，由于TPA与ETTA的反应活性较低，采用冰醋酸浓度为0.1 mol/L的DMSO溶液配制均相预聚溶液。

**图4:**
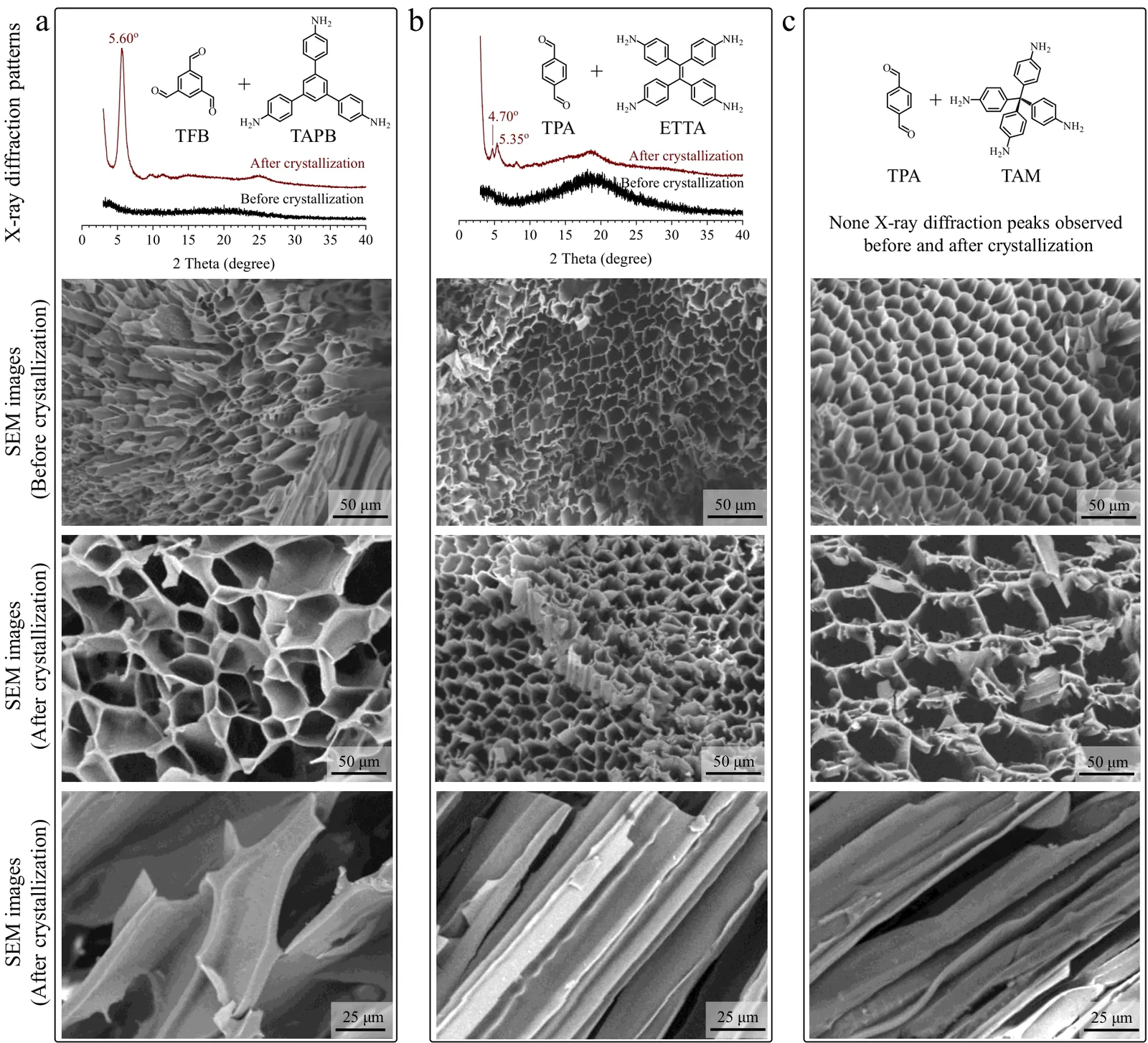
采用不同单体进行冷冻聚合所制备的聚合物整体材料及其晶化后材料的粉末X射线衍射谱图和SEM图 a. TAPB-TFB polymer （before crystallization） and TAPB-TFB COF （after crystallization） synthesized from TFB and TAPB monomers； b. ETTA-TPA polymer （before crystallization） and ETTA-TPA COF （after crystallization） synthesized from TPA and ETTA monomers； c. TAM-TPA polymers （before and after crystallization） synthesized from TPA and TAM monomers.

TFB与TAPB聚合形成的无定形聚合物（图4a，晶化前）在一定程度上呈现定向通孔结构，与ETTA-TPA（图4b）和TAM-TPA（图4c）体系所形成的聚合物骨架不同的是，该聚合物骨架横向之间的黏结较为明显，这可能与TFB与TAPB较快的反应速率和较高的交联度有关，导致在室温配制预聚溶液时就有寡聚物快速生成，从而影响定向冷冻条件下DMSO晶体模板的形成。晶化处理后的整体材料保留了通孔结构，且具有TAPB-TFB COFs材料的特征衍射峰（2*θ=*5.60^o^、9.60^o^、11.38^o^）^［17］^，特征有序孔径为1.18 nm，比表面积为885 m^2^/g，低于采用1，4-二氧六环为溶剂所制备的TAPB-TFB COFs整体材料（1 054 m^2^/g，表1）。

**表1:** 溶剂热法制备的COF粉末材料与冷冻辅助法所制备整体材料的孔结构比较

Monolith	Traditional solvothermal method^*^		Dimethyl sulfoxide crystal as a template （this work）		1，4-Dioxane crystal as a template^**^	
	Surface area/（m^2^/g）	Pore widths/nm	Surface area/（m^2^/g）	Pore widths/nm	Surface area/（m^2^/g）	Pore widths/nm
COF-LZU1	410	1.20	695	1.20	621	1.20
TAPB-TFB COF	1232	1.70	885	1.18	1054	1.18
ETTA-TPA COF	1429	1.17，2.51	1014	1.17，2.51	1530	1.18，2.51
COF-300	1360	0.72	no data	no data	754	0.93

* COF-LZU1^［20］^，TAPB-TFB COF^［21］^，ETTA-TPA COF^［17］^，COF-300 synthesized from TAM and TPA monomers^［22］^. ** Ref. ［^17^］.

TPA与ETTA、TPA与TAM两种体系所制备的无定形聚合物（图4b和4c，晶化前）均呈现出规整的定向通孔结构，充分验证了醛-胺冷冻聚合反应方法制备具有该类孔结构的整体材料的普适性。通过晶化处理，两种整体材料均较好地保留了通孔结构，其中，TAM-TPA聚合物与上述的TFB-PDA聚合物类似，晶化处理导致其内壁出现毛刺结构。粉末X射线衍射谱图和氮气物理吸附测试表明，TPA与ETTA体系能够形成晶态ETTA-TPA COFs整体材料，比表面积为1 014 m^2^/g，而TAM-TPA未能形成晶态的COF-300整体材料。在相同的晶化条件下，采用1，4-二氧六环晶体作为模板所制备的TAM-TPA可成功转化为COF-300晶态材料^［17］^。两种溶剂晶体模板法的不同点在于DMSO与晶化处理所采用的溶液体系不兼容，需要对无定形聚合物整体材料进行洗涤和干燥等预处理，这可能会导致未反应单体及寡聚物的流失，从而影响晶态结构的形成。

## 3 结论

本研究以低温条件下形成的针状DMSO晶体为模板，成功制备了具有定向通孔结构的COFs整体材料，并验证了“先冷冻成型，再高温晶化”两步法的普适性。相较于1，4-二氧六环为模板所制备的材料，采用DMSO晶体模板构筑的整体材料呈现出更为均匀规整的定向通孔结构，表明溶剂模板的选择对形貌调控具有关键影响。此外，相较于已报道的基于粉末COFs与高分子复合的方法，醛-胺冷冻聚合反应策略制备纯COFs整体材料有效避免了复合材料中比表面积降低、孔道堵塞等问题。尽管近年来新型拓扑结构COFs及其分离应用研究不断涌现，但针对COFs材料宏观形貌与微观形貌的调控手段仍发展缓慢。基于此，本工作聚焦于具有微米级通孔的COFs整体材料的创制方法，以期能为从事色谱柱分离及固相萃取领域的科研工作者提供有益借鉴。
